# A Case of Takotsubo Cardiomyopathy Diagnosed After Postponement of Surgery Due to Hypotension and Electrocardiogram Abnormality Upon Entering the Operating Room

**DOI:** 10.7759/cureus.25389

**Published:** 2022-05-27

**Authors:** Kana Kikuchi, Takao Kato, Kaoru Koyama

**Affiliations:** 1 Anesthesiology, Saitama Medical Center, Kawagoe, JPN

**Keywords:** normalcy bias, active cardiac condition, noncardiac surgery, postponement of surgery, perioperative cardiovascular evaluation and management, takotsubo cardiomyopathy

## Abstract

Takotsubo cardiomyopathy (TCM) is a rare disease that is difficult to diagnose. We experienced a case that developed just before surgery. A woman in her 80s with no complications except hypertension was scheduled for colon cancer surgery. Although she was asymptomatic, after entering the operating room, her surgery was canceled due to unexplained hypotension and ST-segment elevation on the electrocardiogram monitor. Emergency coronary angiography was performed immediately, and the presence of TCM was revealed. Her surgery was therefore performed after the improvement in her cardiac function. Once a patient is in the operating room, the normalcy bias kicks in and it becomes difficult to decide to stop the surgery. However, even at this time, it is important to stop the induction of anesthesia if there is any abnormality and to make a differential diagnosis based on the possible development of a serious disease, as seen in this case.

## Introduction

Perioperative Takotsubo cardiomyopathy (pTCM) has been suggested to occur in one in 6,700 patients and at a rate of 5% before induction of anesthesia [1]. However, most cases are detected preoperatively at the time of admission, and very few (0.1%) are suspected to occur after admission [1]. Compared to non-perioperative TCM, pTCM is more common in males and younger patients, has a lower likelihood of chest pain and electrocardiogram (ECG) abnormalities and may have a lower ejection fraction (EF) and higher mortality [1]. In the case reported here, coronary artery disease was suspected due to hypotension and ECG abnormalities before induction of anesthesia, and TCM was finally diagnosed.

Once the patient entered the operating room, normalcy bias was activated, making the decision to abort difficult [2], but a close examination suspected coronary artery disease, which led to a prompt diagnosis. After treatment, the patient underwent reoperation, which had been postponed, and safe perioperative management was achieved.

## Case presentation

The patient was an 84-year-old female with a height of 137 cm and weighed 42 kg. She originally presented with dyspepsia, and laparoscopic sigmoid colon resection was scheduled for a subsequent diagnosis of sigmoid colon cancer. She was taking candesartan 8 mg/day and amlodipine 5 mg/day for hypertension, and systolic blood pressure was controlled at about 120 mmHg. She had never smoked and she occasionally drank alcohol. Her personality indicated an excessive concern about decency and appearance in public.

Preoperative 12-lead ECG and chest radiography showed no abnormal findings. Transthoracic echocardiography (TTE) showed a left ventricular EF (LVEF) of 67%, LV internal diameter shortening of 36%, LV wall motion within normal limits, LV end-diastolic diameter (EDD)/end-systolic diameter (ESD) of 34/22 mm, peak velocity blood flow from left ventricular relaxation in early diastole (E-wave) of 59 cm/sec, peak velocity flow in late diastole caused by atrial contraction (A-wave) of 82 cm/sec, the ratio of E-wave to A-wave (E/A ratio) of 0.7, ventricular septal wall (VSW) thickness/LV posterior wall thickness of 9/10 mm, and no hypertrophy, mass, or valve disease. Based on the American Society of Anesthesiologists Physical Status class II, we decided to perform elective surgery.

Blood pressure at admission was 127/73 mmHg and heart rate was 105 beats/min. On the morning of the surgery (six hours before entering the operating room), blood pressure was 70/35 mmHg and heart rate was 100 beats/min. The ward nurse discontinued antihypertensive medication according to clinical path instructions, but this was not noted in the medical record. Three hours before entering the operating room, blood pressure was 90/52 mmHg and heart rate was 80 beats/min. The patient walked into the operating room without reporting the early morning hypotension to the attending physician or anesthesiologist. This hypotension was discovered in the entry report, but the attending physician judged that the patient was operable since she had no subjective symptoms because they determined that it was a vagal reflex caused by laxative defecation.

**Figure 1:**
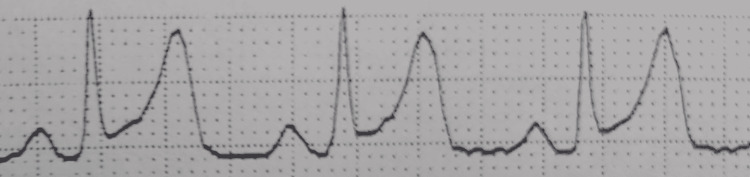
Electrocardiogram monitored in the operating room ST-segment elevation and T-wave hyperintensity were observed in II.

**Figure 2:**
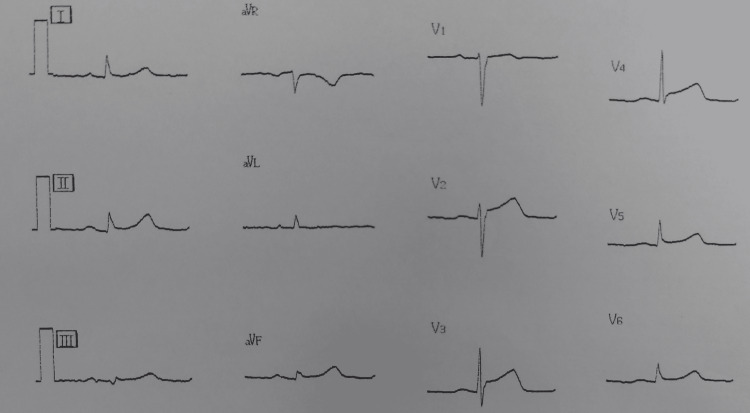
ST-segment elevation and T-wave hyperintensity were observed in II. Significant ST-segment elevation was observed in I, II, aVF, and V2-6.

Immediately after entering the operating room, blood pressure was 91/57 mmHg, heart rate was 82 beats/min, and peripheral oxygen saturation (SpO2) was 95% under room air, Although the patient had no subjective symptoms, we performed a 12-lead ECG (Figure 1) because of significant ST-segment elevation in I, II, aVF, V2, V3, V4, V5, and V6 on the monitor ECG (Figure 2). In addition, we reconfirmed the blood pressure trend from before admission and suspended induction of anesthesia.

Blood tests showed a white blood cell count (WBC) of 11400 /μL, hemoglobin (HGB) 8.4 g/dL, creatinine kinase (CK) 655 IU/L, CK myocardial band (CK-MB) 20 IU/L (normal: 0-25 IU/L) and cardiac troponin I 6.36 ng/mL (standard value: 0-0.09). The TTE showed a high degree of global wall hypokinesia at the apex, although the base was moving.

**Figure 3:**
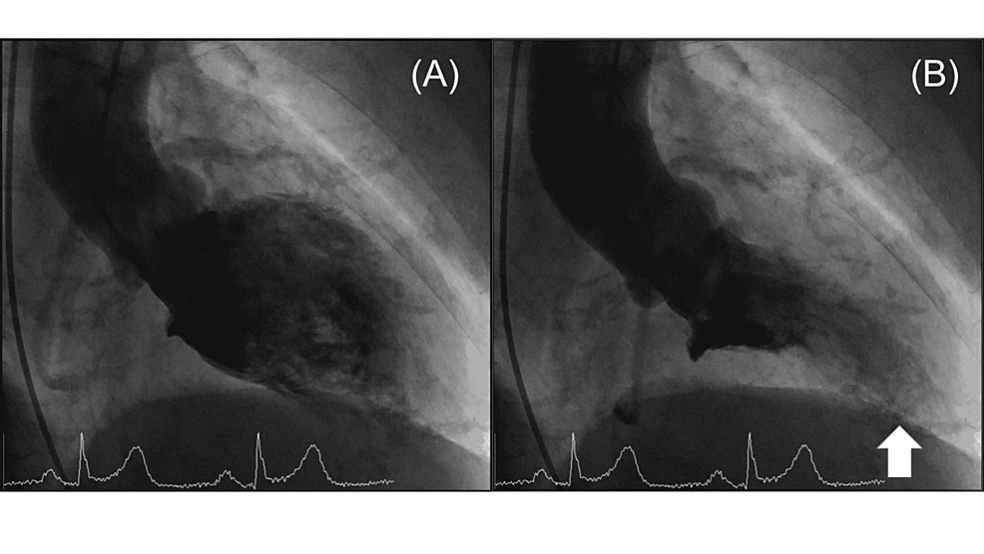
Left ventriculography images (A) Diastolic, (B) Systolic. There were findings of left ventricular apical ballooning during systole.

After consultation with the surgical team, we decided to cancel the operation. Based on examination findings, acute coronary syndrome (ACS) was suspected and the patient was referred to the cardiology service, where emergency coronary angiography (CAG) was performed. The CAG showed no significant stenosis of the coronary arteries. Based on findings of apical ballooning (Figure 3), TCM was diagnosed and the patient was placed in the cardiac care unit. Three hours later, blood samples showed WBC 10400/μL, HGB 7.8 g/dL, CK 538 IU/L, CK-MB 16 IU/L, and cardiac troponin I 5.33 ng/mL. At 18 hours, CK was 271 IU/L, and CK-MB 12 IU/L. Thirty-one hours after entering the operating room, brain natriuretic peptide was 100.3 pg/mL.

**Figure 4:**
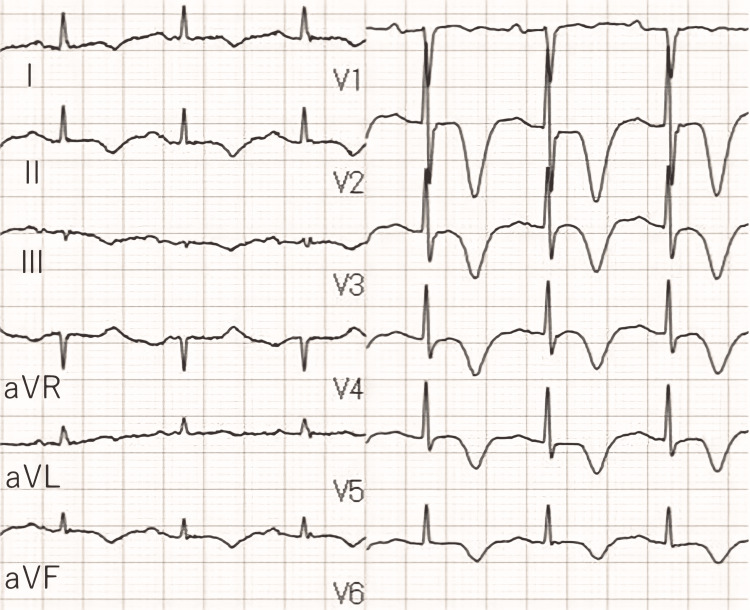
Twelve-lead electrocardiogram at 14 days after onset of Takotsubo cardiomyopathy A negative T wave was still present.

The patient was transferred to a general ward on day four of hospitalization after her general condition improved in the cardiac care unit and was discharged on day 10 without further deterioration. Fourteen days after the onset of TCM, the patient was hospitalized for four days for urinary tract infection (UTI). At the time of admission, the negative T-wave on the ECG was still present (Figure 4).

After confirming the improvement of cardiac function in the outpatient clinic on day 36 after the onset of TCM, the operation was rescheduled for day 51. On day 36, the TTE showed B-mode LVEF of 72%, LV internal diameter shortening of 41%, posterior inferior wall (mid) hypokinesis, LV EDD/ESD of 40/24 mm, E/A ratio of 0.6, and VSW thickness/LV posterior wall thickness of 11/10 mm.

After an outpatient ultrasound confirmed improvement in cardiac function, the patient was scheduled for surgery on day 51. We reminded the ward nurses that if a patient's blood pressure is low, they should not just report it as low, but also report the value to the surgeon. Psychosupportive care including family members was provided to reduce the psychological stress of the surgery. No pre-anesthetic medication was administered. Vital signs on entering were blood pressure 141/77 mmHg, heart rate 86 beats/min, and SpO2 94% under room air. After placing a catheter for thoracic epidural anesthesia, an arterial line was secured under conscious control. The patient was managed under general anesthesia with epidural anesthesia. Preoperative echocardiography showed residual hypokinesis in part of the myocardium, so anesthesia induction was performed with midazolam 3 mg, fentanyl 200 µg, and rocuronium 30 mg. After endotracheal intubation, anesthesia was maintained with sevoflurane and remifentanil 0.1-0.2 µg/kg/min.

Postoperatively, systemic management was performed in the intensive care unit (ICU). Circulation was stabilized with a small dose of noradrenaline. The pain was well managed with continuous epidural anesthesia, and the patient was transferred to a general ward on postoperative day (POD) 1 and discharged on POD 10 without any further problems. She had been coming to our hospital for follow-up of her cancer for five years after surgery, during which time there was no recurrence of cancer or TCM.

## Discussion

The guidelines for perioperative cardiovascular evaluation and management for noncardiac surgery cover noncardiac surgery in patients with cardiac disease [3]. In this case, we examined whether the patient was in an active cardiac condition. On the day of surgery, blood pressure dropped, ECG changed, and there were no subjective symptoms. However, given that the patient was an elderly woman, we considered the possibility of unstable coronary syndromes and determined that a thorough examination was necessary.

A decision to stop surgery is often made by the anesthesiologist, after consultation with the surgeon, regardless of the facility or years of experience [4]. However, in this case, even though the patient had hypotension preoperatively, the information was unknown and the patient entered the operating room. Although the patient was elderly, she had only well-controlled hypertension and was able to walk into the room, which may have enhanced the normalcy bias [2]. However, we strongly suspected an active cardiac condition based on the reconfirmation of information from the 12-lead ECG, and echocardiography, and were able to make an appropriate decision to stop the surgery.

Compared to ST-elevation and non-ST-elevation myocardial infarction, TCM is characterized by lower cardiac troponin I levels and higher BNP. There is still no non-invasive means of definitively differentiating ACS from TCM [5], and a lack of CAG in the diagnosis of TCM may be independently associated with increased in-hospital mortality due to missed ACS [6]. In this case, we were able to reliably exclude ACS by performing CAG in the acute phase.

Cancer patients are at high risk of developing TCM due to emotional and psychosocial stress, and physical stress from surgery and radiotherapy [7], which may have been the main factor in the development of TCM in this case. In the National Inpatient Sample study [8], TCM with concomitant malignancy had significantly higher mortality, length of hospital stay, and total costs, compared to TCM without malignancy. Mortality may also be higher in the short and long term [9]. Therefore, early detection and prompt initiation of appropriate treatment may be useful in reducing mortality and healthcare costs [7].

The pathogenesis of TCM is unknown and spontaneous recovery occurs without complications, but therapy using standard guidelines for heart failure with reduced EF should be used to manage patients with hemodynamic stability [7]. There are no prospective clinical trial data to indicate the optimal duration of therapy, but this should be continued until the systolic function is restored, which usually requires one to four weeks [10,11]. In the present case, reoperation after four weeks was considered, but this took longer due to complications of UTI. There are no clinical data indicating when surgery is possible, but it is preferable to wait for normalization of LVEF, which is also the first goal of treatment [11]. It has also been suggested that the resumption of chemotherapy should follow the normalization of LVEF [10]. In our case, reoperation was scheduled after recovery of LVEF was confirmed by echocardiography.

The rate of recurrence of TCM is estimated to be 1.5% to 4% [11,12] and it is advisable to continue pharmacotherapy indefinitely [13]. Aspirin, beta-blockers, angiotensin-converting enzyme inhibitors, and statins are often prescribed for TCM, and some reports suggest the use of angiotensin II receptor blockers (ARBs) alone or in combination with beta-blockers and statins [14]. Recurrence and in-hospital complications may also be more common in patients with hypertension [12]. Since our case was also associated with hypertension, we considered the possibility of recurrence and additional administration of drugs. However, no aspirin or statins were required because there was no significant stenosis on CAG, and ARBs and beta-blockers were not used because blood pressure and heart rate were both well controlled.

We considered pre-medication to reduce stress during reoperation, but we finally substituted this with supportive care, including family members, in this case. Since preoperative echocardiography showed residual hypokinesis in part of the myocardium, a hemodynamic arterial pressure line was inserted before induction of anesthesia. General anesthesia was induced carefully with midazolam to minimize circulatory changes associated with anesthesia induction. Postoperatively, the patient remained in the ICU for strict systemic management until postoperative day one.

## Conclusions

We experienced a case of TCM diagnosed after the postponement of surgery but occurred from the day before to the day of surgery. This case reaffirms the importance of working closely with ward nurses and the attending physician to stop the induction of anesthesia if there is any abnormality, even after the patient enters the operating room, and to make a differential diagnosis based on the possibility of a serious disease.
